# Quantum dot-aluminum phthalocyanine conjugates perform photodynamic reactions to kill cancer cells via fluorescence resonance energy transfer

**DOI:** 10.1186/1556-276X-7-386

**Published:** 2012-07-12

**Authors:** Lei Li, Jin-Feng Zhao, Nayoun Won, Ho Jin, Sungjee Kim, Ji-Yao Chen

**Affiliations:** 1 State Key Laboratory of Surface Physics, Department of Physics, and Key Laboratory of Micro and Nano Photonic Structures (Ministry of Education), Fudan University, Shanghai,, 200433, People's Republic of China; 2 Department of Chemistry, Pohang University of Science and Technology (POSTECH), San 31, Hyoja-Dong, Nam-Gu, Pohang, Gyeong-Buk,, 790-784, South Korea

**Keywords:** Quantum dot, Aluminum phthalocyanine, Fluorescence resonance energy transfer, Photodynamic therapy of cancers.

## Abstract

Sulfonated aluminum phthalocyanines (AlPcSs), commonly used photosensitizers for photodynamic therapy of cancers (PDT), were conjugated with amine-dihydrolipoic acid-coated quantum dots (QDs) by electrostatic binding, achieving 70 AlPcSs per QD. The AlPcS-QD conjugates can utilize the intense light absorptions of conjugated QDs to indirectly excite AlPcSs producing singlet oxygen via fluorescence resonance energy transfer (FRET), demonstrating a new excitation model for PDT. The AlPcS-QD conjugates easily penetrated into human nasopharyngeal carcinoma cells and carried out the FRET in cells, with efficiency around 80%. Under the irradiation of a 532-nm laser, which is at the absorption region of QDs but not fit for the absorption of AlPcSs, the cellular AlPcS-QD conjugates can destroy most cancer cells via FRET-mediated PDT, showing the potential of this new strategy for PDT.

## Background

Photodynamic therapy (PDT) has been established as a new treatment modality for cancers during the past two decades. The principle of this modality is that a photosensitizing drug can preferably accumulate in the cancer region and produce singlet oxygen (^1^O_2_) when excited with light of appropriate wavelengths to destroy the lesion [1]. Therefore, the efficiency of cancer inactivation is closely correlated to the light absorption of used photosensitizers (PSs). However, the light extinction coefficients of PSs are generally low, including those officially approved PSs such as Photofrin and metal phthalocyanines [2-4]. Recently, the nanotechnology with different kinds of nanoparticles has been increasingly developed, and the related applications have been expanded quickly [5-10]. The suggestion of combining nanotechnology with PDT has been proposed, and related works have been tested [11-18]. Based on the high light absorption coefficients of quantum dots (QDs), Samia et al. firstly proposed a new idea of PS-QD conjugate model, in which the QD absorbs light and then functions as a donor to transfer the energy to PS via fluorescence resonance energy transfer (FRET). They prepared QD-phthalocyanine (Pc4) conjugates in organic solution, and these conjugates achieved the high ^1^O_2_ production with the way of FRET [19]. The other groups including our group then developed different kinds of QD-PS conjugates in aqueous solutions for their further applications in biological systems [5,20,21]. Since the light extinction coefficients of QDs are one order of magnitude larger than that of PSs, the conjugation of PS-QD seems a potential way to increase the PDT effect. However, the biological system is much complicated as compared to the pure solution. So far, the FRET-mediated PDT by PS-QD conjugates has not been achieved in living systems, leaving a doubt whether the PS-QD conjugates can really work in PDT. Regarding the application in biological systems such as in living cells, at least two problems should be overcome. Firstly, when conjugates pass through the plasma membranes entering the living cells, they should keep their conjugate form, which requires a stable binding between PS and QD in the conjugate. Secondly, the PS-QD conjugates are also required to maintain their physical properties in the cellular environment for carrying out the FRET. In this work, the stable conjugates of QDs with sulfonated aluminum phthalocyanines (AlPcSs) were prepared by electrostatic binding. These conjugates can load AlPcS to penetrate into cancer cells easily and then perform the FRET effectively in cellular environments. The conjugates in cells demonstrated for the first time that they can kill cancer cells via FRET-mediated PDT.

## Methods

### Preparation of amine-DHLA-coated CdSe/CdS/ZnS QDs

**Figure 1:**
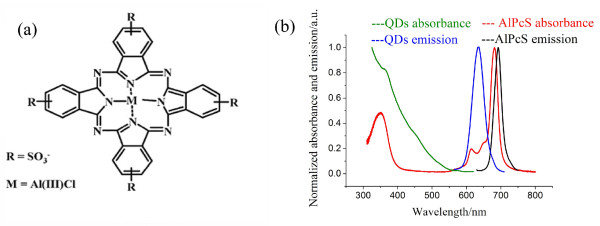
**The conjugates and their absorption and photoluminescence spectra.** (**a**) Molecular structure of tetrasulfonated aluminum phthalocyanine (AlPcS). (**b**) The absorption and emission spectra of QDs and AlPcSs in aqueous solutions.

Water-soluble amine containing dihydrolipoic acid derivatives (amine-DHLA)-QDs were prepared according to our previous report [22]. The CdSe/CdS/ZnS (core/shell/shell) QDs were synthesized firstly [23] and were then exposed to an excess amount of amine-DHLA for rigorous surface exchange to form the amine-DHLA-QDs. Due to the positive charge on the amine terminal, these QDs became dispersible in aqueous media. The absorption and photoluminescence (PL) spectra of these QDs are shown in Figure 1b.

### Preparation of AlPcS-QDs

The photosensitizer AlPcS (Frontier Scientific, Inc, Logan, UT, USA) could have the maximum four negative charges in its four benzene rings (Figure 1a), so that they can bind on the surfaces of QDs by an electrostatic force. The absorption and fluorescence spectra of AlPcSs in aqueous solution, measured by a spectrophotometer (Hitachi F-2500, Hitachi, Tokyo, Japan), are shown in Figure 1b. To prepare the AlPcS-QD conjugates, the mixture groups of AlPcSs and QDs with different molar ratios were made and stirred overnight in the dark at room temperature (25 °C). These mixture solutions were then centrifuged at 10,000 rpm for 30 min to separate the AlPcS-QD conjugates in the bottom of the centrifuging tubes and the unbound AlPcSs in the supernatants. The harvested AlPcS-QD conjugates were re-suspended in aqueous solution for further experiments. The FRET efficiencies of these obtained complexes were measured respectively to find out an optimal molar ratio for AlPcS-QD conjugates with the best FRET effect. From the obtained results, an optimal molar ratio of AlPcSs to QDs was found to be about 100:1.

#### Cell culture

The human nasopharyngeal carcinoma cells (KB cells) were obtained from the cell bank of Shanghai Science Academy, Shanghai, China. The cells were seeded into culture dishes containing DMEM medium with 10% calf serum, 100 units/ml penicillin, 100 μg/ml streptomycin and 100 μg/ml neomycin, and incubated in a fully humidified incubator at 37 °C with 5% of CO_2_. When the cells reached 80% confluence with normal morphology, AlPcS-QDs were added and incubated in an incubator for 50 min. After incubation, these cells were washed three times with phosphate buffered saline (PBS) to remove unassociated AlPcS-QDs, and then, these cell samples were ready for further experiments.

### Imaging measurements of AlPcS-QDs in cells

The fluorescence images of free QDs, free AlPcSs and AlPcS-QD conjugates in KB cells were measured in a laser scanning confocal microscope (LSCM) (FV300, IX71, Olympus Microscopy, Tokyo, Japan) excited by a 405-nm laser. In the measurements, the QD's PL was measured in detection channel 1 of the LSCM with a band-pass filter of 585 to 640 nm, and the fluorescence signal of AlPcSs was recorded in detection channel 2 with a 670-nm long-pass filter. Differential interference contrast (DIC) images were obtained simultaneously in a transmission channel to exhibit the cell morphology. A water immersion objective (×60) and a matched pinhole of LSCM were used in experiments.

### QD's PL lifetime measurements by TCSPC

Time-correlated single photon counting (TCSPC) is a common method to detect the fluorescence lifetime [24,25]. In the experiments, with the excitation of a 70-ps laser pulse from a 405-nm diode laser (EPL405, Edinburgh Instruments, Livingston, UK), the PL decay courses of QDs in aqueous solutions or cells were measured by a PMT (R928P, Hamamatsu, Yokohama, Japan) with a band-pass filter of 585 to 640 nm in the TCSPC (TCC900, Edinburgh Instruments). The obtained PL decay curves can be fitted with a multi-exponential decay, as described in Equation 1.

(1) $$I(t)=\sum_{i=1}^{n}\alpha_{i}\exp\left(-\frac{t}{\tau_{i}}\right)$$

where *τ*_*i*_ and α_*i*_ represent the decay constant and amplitude of each exponential component. The average lifetime $\bar{\tau }$ can be obtained then according to Equation 2 [26].

(2) $$\bar{\tau }=\frac{\sum_{i=1}^{n}\alpha_{i}\tau_{i}^{2}}{\sum_{i=1}^{n}\alpha_{i}\tau_{i}}$$

When the *τ*_0_ and *τ*, the average lifetime of free QDs and that of conjugated QDs in AlPcS-QDs, were obtained, the FRET efficiency of AlPcS-QD conjugates was calculated by Equation 3 [27,28].

(3) $$E=1-\frac{\tau }{\tau_{0}}$$

We found that two exponential components are good enough to fit the measured decay curves here. With the numerous measurements, the average PL lifetimes of QDs in different cases were determined.

### Cytotoxicity assay

The 3-(4,5-dimethylthiazol-2-yl)-2,5-diphenyltetrazolium bromide (MTT) tetrazolium reduction assay was used to measure PDT damaging of AlPcS-QDs on cells. The KB cells with the concentration of 3 × 10^4^ cells/ml were equally seeded in each well of a 96-well flat-bottom tissue culture plate and allowed to attach to the plate overnight. Then, 5 μM AlPcS, 0.05 μM QDs and 0.05 μM AlPcS-QDs were added into different wells for 50-min incubation, respectively. After the incubation, the cells were washed three times with PBS to remove the unassociated compounds, and added with the fresh medium. These cells were then irradiated by a halogen lamp or a 532-nm laser at different times. After illuminations, the cells were incubated in an incubator for 24 h, and then, 10-μl MTT solution (5 mg/ml) was added into each well for incubation for 1.5 h. Finally, the 96-well plates were put into an iEMS analyzer (Laboratory Systems Group Pty Ltd., Kilsyth, Victoria, Australia) to measure optical densities (O.D) at 450 nm for each well. The cell viability in each well was determined by comparing their O.D value with that of untreated control cells in some wells in the same plate. All results were presented as the mean ± standard deviation (SD) from three independent experiments with four wells in each.

### ROS assays

Reactive oxygen species (ROS), such as singlet oxygen (^1^O_2_) and free radicals of oxygen molecules, play an important role in PDT. When the conjugated AlPcSs in AlPcS-QDs were excited via FRET, they could emit fluorescence in one way and produce ^1^O_2_ (excited state of O_2_) by transferring the energy to the surrounding oxygen molecules in the other way. 1,3-Diphenylisobenzofuran (DPBF), a sensitive probe of ROS [29], was used to detect the ROS produced by a FRET way of AlPcS-QDs under irradiation at 532 nm. The DPBF can be quickly oxidized by ROS to become o-dibenzoylbenzene, leading to a degradation of DPBF with reduced fluorescence intensity. The fluorescence of DPBF was measured in a spectrometer (F-2500, Hitachi) with the excitation at 405 nm. In the experiment, the DPBF (12 μM) were mixed with AlPcSs (10 μM), QDs (0.1 μM) and AlPcS-QDs (0.1 μM) in aqueous solutions, respectively, and then, these samples were irradiated by a 532-nm laser (16 mW) for different times such as 20 s, 40 s, etc. The fluorescence intensity decrement of DPBF is usually used as the indicator of the ROS production during the photosensitization process of PSs. The degradation rate of the DPBF with the irradiation time is proportional to the ROS yield and, thus, can be used to evaluate the relative ROS yields for different compounds. Sodium azide (NaN_3_), a specific ^1^O_2_ scavenger, was also used in the experiment to confirm the ^1^O_2_ production in the FRET process of AlPcS-QDs [30].

## Results and discussion

**Figure 2:**
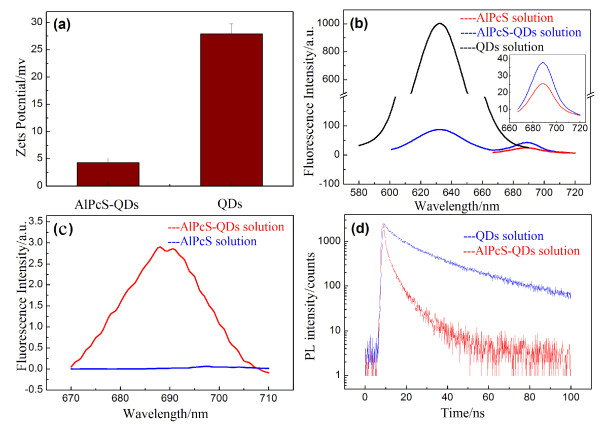
**Time-correlated single photon counting measurements.** (**a**) Zeta potential measurements for AlPcS-QDs and QDs. (**b**) Emission spectra of the QDs (0.05 μM), AlPcSs (5 μM) and AlPcS-QDs (0.05 μM) in aqueous solutions. Excitation, 405 nm. (**c**) Fluorescence comparison of free AlPcS and AlPcS-QDs in aqueous solutions under the excitation at 532 nm. (**d**) PL lifetime curves of the conjugated QDs and free QDs in aqueous solutions excited by a 405-nm ps laser and measured by TCSPC with a 585- to 640-nm band-pass filter.

AlPcS possesses four negative charges on its four benzene rings (Figure 1a). The CdSe/CdS/ZnS QDs are covered with amine-DHLA, so that they have positive charges on their surfaces [22]. These QDs with the PL band of 630 nm were used to link with AlPcSs in this work because the 630-nm PL band of QDs (donor) is well-overlapped with the absorption band of AlPcSs (acceptor), satisfying the FRET principle (Figure 1b). The zeta potential of +26 mV confirmed that these QDs have the positive charges on surfaces (Figure 2a). After conjugation of QDs with AlPcSs in aqueous solution at the molar ratio of 1:100, the AlPcS-QD conjugates were formed by electrostatic binding. The conjugate solution was then centrifuged at 10,000 rpm for 30 min to harvest the conjugates. The content of unconjugated free AlPcSs in the supernatant was determined by comparing their fluorescence intensity with that of the initial AlPcS amount before the conjugation. About 30% AlPcSs were left in the supernatant, which means that 70% AlPcSs were bound to QDs, and thus, the conjugation rate was about 70 AlPcSs per QD. Since the AlPcSs have been linked on the QD surfaces, the zeta potential of AlPcS-QDs decreased to +4 mV (Figure 2a). Figure 2b shows the emission spectra of free QDs (0.05 μM), free AlPcSs (5 μM) and AlPcS-QDs (0.05 μM) in aqueous solutions under the excitation at 405 nm. The emission intensity (630 nm) of conjugated QDs greatly decreased, and the emission intensity (690 nm) of AlPcSs moiety of AlPcS-QDs increased, as compared with that of unconjugated QDs or AlPcSs, demonstrating a typical FRET model [5,20,21]. The FRET effect was further shown by an emission comparison between conjugated AlPcSs and free AlPcSs under the excitation at 532 nm (Figure 2c). No emission could be recorded for free AlPcSs because AlPcSs almost have no absorption at 532 nm (Figure 1b). However, the obvious emission band occurred in the AlPcS-QD solution under the excitation at 532 nm, which must have resulted from the effect of FRET. The QDs in conjugates absorbed the excitation light, and then, these excited QDs transferred the energy to conjugated AlPcSs to make AlPcSs excited, resulting in AlPcS emissions. The FRET efficiency of QDs to AlPcSs in conjugates was estimated by the PL lifetime measurements using the method of TCSPC with the excitation of a PS pulse laser (405 nm). As shown in Figure 2d, the PL lifetime of conjugated QDs remarkably reduced, as compared with that of free QDs. The average PL lifetimes for them are 3.88 ± 0.09 ns and 33.3 ± 0.5 ns, so that the FRET efficiency (*E*) of conjugates reaches 88.8%.

**Figure 3:**
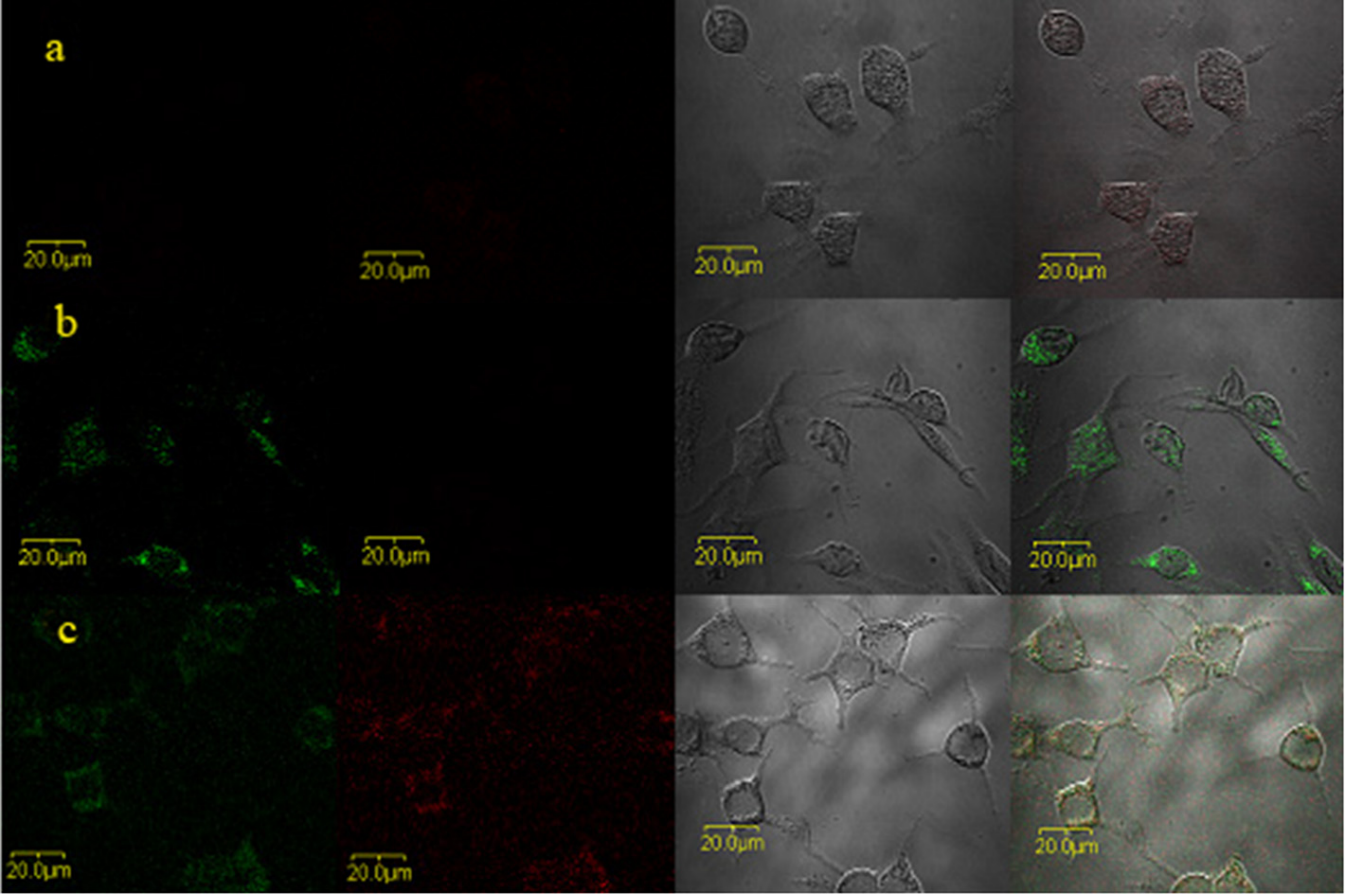
**Confocal images of AlPcSs (a), QDs (b) and AlPcS-QDs (c) in KB cells.** Columns from left to right: (1) PL images detected in the 585- to 640-nm channel, (2) fluorescence images measured in the channel with a 670-nm long-pass filter, (3) DIC images and (4) merged images.

The AlPcSs are famous PSs possessing high ^1^O_2_ yield, but the negative charges on its benzene rings obstruct the fast cellular uptake because the cell surface is negative-charge-dominated also. After the conjugation of AlPcSs with QDs, the conjugate surface still retained some positive charges (+4 mV, see Figure 2a), which would facilitate the cellular uptake of AlPcS-QDs. As shown in Figure 3a,b, a little AlPcSs entered the cells after 50-min incubation with free AlPcSs (5 μM), whereas the cellular uptake for free QDs (0.05 μM) was so obvious. The confocal fluorescence images of cellular AlPcS-QDs became pronounced too after 50-min incubation, indicating that the AlPcS-QDs can quickly penetrate into cells. With the measurements of two channels, the 585- to 640-nm channel for QDs and the 670-nm long-pass channel for AlPcSs, the cellular images of conjugated QDs and AlPcSs were obtained respectively (Figure 3c). The exact overlapping of these two images (Figure 3c) convinces that the QDs and AlPcSs are still bound together in cells, demonstrating that the electrostatic binding of AlPcS-QD is stable in the cellular environment.

**Figure 4:**
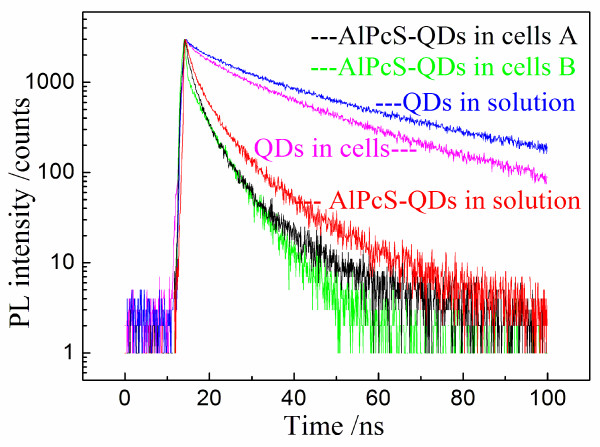
**PL lifetime curves of the conjugated QDs and free QDs in aqueous solutions and KB cells.** Excited by a 405-nm ps laser and measured by TCSPC with a 585- to 640-nm band-pass filter.

Since the AlPcS-QDs distribute in the cytoplasm of cells, the effects of the cellular environment on their binding and their FRET property need to be checked. As shown in Figure 3b,c, the PL images of free QDs and conjugated QDs in cells have been obtained, respectively, and then, some micro-regions in those images were selected to measure the PL lifetimes. The average PL lifetime of free QDs in cells was measured as 24.8 ± 1.5 ns, slightly shortened as compared with that of free QDs in aqueous solution, reflecting an influence of the cellular environment (Figure 4). When QDs entered the cells, some negatively charged bio-molecules in cytosol may adhere on QD surfaces, playing a FRET-like role to dissipate a small-part energy, resulting in a slight reduction of PL lifetime. The PL lifetime of cellular AlPcS-QDs greatly decreased with a feature of increased fluctuation in different micro-regions. The average PL lifetime of conjugated QDs with the SD in cells is 4.15 ± 1.13 ns (Figure 4). Therefore, the FRET efficiency of cellular AlPcS-QDs still reached 84%, according to Equation 3 and taking the PL lifetime of free QDs in cells as the reference. Regarding the average value, the fluctuation extent of PL lifetimes of cellular AlPcS-QDs is about 25%. In the cytoplasm of cells, the distribution of electric field is complex in different micro-regions due to a complicated distribution of charged bio-molecules. This micro-environment of electricity may affect the binding extent of QD and AlPcSs for cellular AlPcS-QDs, resulting in a variation of PL lifetimes in different micro-regions. However, the average FRET efficiency of cellular AlPcS-QDs is still in a high level, so that the PDT damaging to cells could be conducted via FRET.

**Figure 5:**
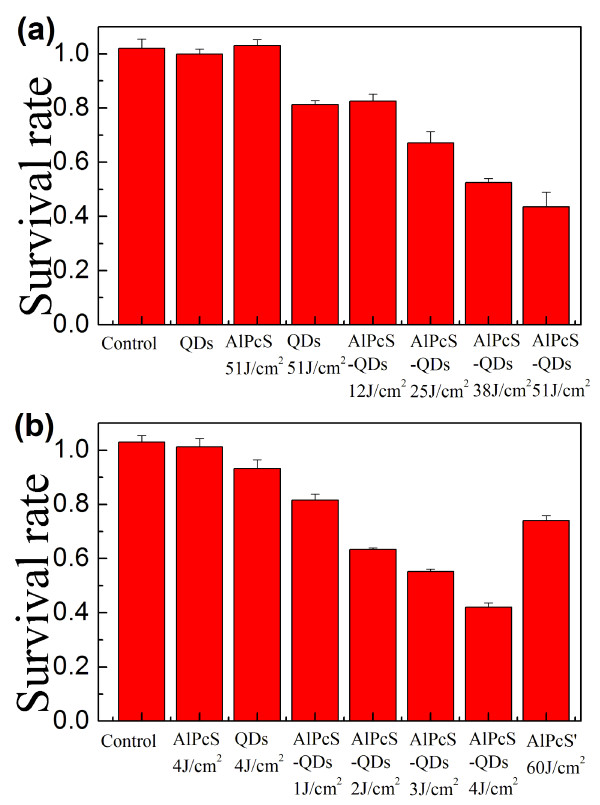
**The PDT damaging on KB cells, measured by a MTT assay.** Except the control groups, cells were incubated with free AlPcSs (5 μM), QDs (0.05 μM) or AlPcS-QDs (0.05 μM) for 50 min, respectively, followed by the irradiation of a 532-nm laser (**a**) or a halogen lamp (**b**). In AlPcS' column in Figure 4b, cells had been incubated with AlPcSs (5 μM) for 3 h and were then irradiated.

The MTT assay is a common method to evaluate the cell viability in a large number of cells [31]. The cell damaging after different treatments and/or different dose irradiations was quantitatively measured, as compared with that of untreated control cells. When cells were incubated with QDs (0.05 μM) or AlPcS (5 μM) for 50 min, no detectable damage could be found in these cells without light irradiations. Moreover, as shown in Figure 5a, after an irradiation at 532 nm with a high light dose of 50 J/cm^2^, about 20% cells were dead in the group of free QD incubated cells, and still, no cell damage could be found for free AlPcS incubated cells. QDs may photo-produce free radicals under a high-dose irradiation, resulting in a slight cell damaging [32]. For the free AlPcS incubated cells, the low cellular accumulation of AlPcSs after 50-min incubation (see Figure 3a) and little absorption of AlPcSs at 532 nm led to a very low toxicity to cells. For the group of AlPcS-QDs incubated cells, the irradiation at 532 nm was expected to carry out the FRET-mediated PDT to kill the cancer cells. As shown in Figure 5a, when cells had been incubated with AlPcs-QDs for 50 min and then irradiated by a 532-nm laser, the cell damage became significant with the statistical *p* value much smaller than 0.01, and the cell death was proportional to the irradiation dose showing a typical light-dose-dependent PDT mode. This result demonstrates for the first time that the AlPcS-QD conjugates can perform the photodynamic reaction via FRET to kill cancer cells.

AlPcSs have an absorption band at around 675 nm (630 to 700 nm) in the visible region, but QDs have broad absorptions in the whole visible area (Figure 1b). A light source with a broad wavelength region may increase the PDT effect of AlPcS-QDs to cancer cells because the AlPcS moiety in conjugates can directly absorb the light around 675 nm to initiate the PDT, and the QD moiety can absorb the light of the other wavelengths shorter than the 675-nm band to conduct a FRET-mediated PDT, resulting in a combination effect to increase the PDT efficiency finally. A 150-W halogen lamp combined with a heat-isolation filter was used here to check the PDT effect of AlPcS-QDs further. The emitting spectrum of this light source is from 400 to 800 nm. Under the irradiation of this white light, the PDT efficiency of conjugates increased obviously (Figure 5b). With a 4-J/cm^2^ irradiation dose of this white light, AlPcS-QDs damaged most cancer cells. Therefore, fully utilizing the absorptions of both QDs and AlPcSs in conjugates is probably a good strategy for PDT. To further compare the PDT effect of AlPcS-QDs with that of free AlPcS, cells were incubated by free AlPcS (5 μM) with a prolonged incubation time (3 h) to allow more AlPcSs penetrating into the cells, and then, these cells were irradiated by the white light. As shown in Figure 5b, even at a high light dose, only about 30% cell death could be conducted in this case.

**Figure 6:**
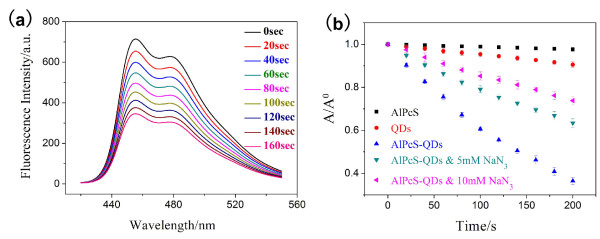
**DPBF used to check the ROS produced in the FRET process of AlPcS-QDs.** (**a**) Fluorescence intensity decreasing of DPBF (12 μM) in AlPcS-QD (0.1 μM) aqueous solution with the irradiation time of a 532-nm laser (16 mW). Excitation, 405 nm. (**b**) The comparison of DPBF photo-degradations of AlPcS-QD (0.1 μM), AlPcS (10 μM) and QD (0.1 μM) aqueous solutions under the irradiation at 532 nm. The protection effect of NaN_3_ (the quencher of ^1^O_2_) on DPBF degradation was demonstrated.

It is well known that the ^1^O_2_ is the reactive agent of AlPcS PDT. The reactive agent could still be the ^1^O_2_ for AlPcS-QD-induced PDT via FRET, but this deduction needs to be proven. The DPBF, a sensitive probe of reactive oxygen species, was used to check the ROS produced in the FRET process of AlPcS-QDs. The oxidation of DPBF by ROS leads to a decrease of DPBF fluorescence, and the reducing rate of DPBF fluorescence is usually used to measure the ROS yield [29]. In the experiment, the QDs (0.1 μM), AlPcSs (10 μM) or conjugates (0.1 μM) were mixed with DPBF (12 μM), respectively, and then, the mixtures were in turn irradiated by a 532-nm laser (16 mW). Figure 6a shows a decreasing course of DPBF fluorescence with the irradiation time (in seconds) for AlPcS-QDs. The comparison of DPBF degradations by free QDs, free AlPcSs and AlPcS-QDs were shown in Figure 6b. No decrement of DPBF fluorescence could be found for the AlPcSs (10 μM) group, which is reasonable because AlPcSs have no absorption at 532 nm. A slight decrement of DPBF fluorescence suggests that QDs (0.1 μM) alone could induce a small production of ROS under the irradiation at 532 nm. The remarkable reduction of DPBF fluorescence indicates that the AlPcS-QDs (0.1 μM) photo-produced a large amount of ROS via FRET. When sodium azide (NaN_3_), a specific ^1^O_2_ scavenger [31], was added into the mixture of AlPcS-QDs (0.1 μM)-DPBF (12 μM) solution, and then this sample was irradiated by a 532-nm laser in the same way, the DPBF fluorescence was obviously protected (Figure 6b), indicating that the reactive species in the FRET-mediated photodynamic reaction of AlPcS-QDs is still ^1^O_2_.

The tissue transparency window is in the region of 650 to 900 nm, so that the wavelengths in this region are optimal for carrying out PDT. However, QD's main absorption region is shorter than 600 nm, which does not match the tissue window wavelengths. Another advantage of QDs can just help them overcome this limitation. QDs have very huge two-photon absorption cross sections (thousands Goppert Mayer (GM)) in the near-infrared (NIR) wavelength region. Therefore, QDs in conjugates can be excited by two-photon excitation (TPE) with a femtosecond NIR laser and then initiate FRET-mediated PDT. Since the two-photon absorptions of PSs are very low (a few GM), the PS-QD conjugates with TPE open a new way for the so-called two-photon PDT. Our next work is already under processing toward this direction.

The main disadvantage of PSs used in PDT is skin phototoxicity because the affinity of PSs to cancers is not high, and a part of PSs remain in the skin. If the drug delivery efficiency to cancers is increased, the drug dose can be decreased, leading to a lower content of drugs in the skin, reducing skin phototoxicity. However, the accumulation of PSs in cancer is a passive targeting. Based on the passive targeting mechanism for cancers *in vivo*, the particles with the size of 3 to 100 nm will be accumulated more in tumors than smaller molecules due to the effect of enhanced permeability and retention. The sizes of current used PSs are all about 1 nm, so that the delivery efficiency to tumors is low. However, the size of the QDs we used is about 3.5 nm, and the sizes of PS-QD conjugates should be a little bit bigger than 3.5 nm. Therefore, with the PS-QD conjugates, better tumor accumulation is expected. The use of PS-QD conjugates may also benefit drug delivery and decrease skin phototoxicity.

## Conclusions

AlPcS-QD conjugates were stable not only in solutions but also in cellular environments. The cellular AlPcS-QDs achieved FRET in living cells with an efficiency of 84%, reflecting that the AlPcS-QDs possess the ability to carry out PDT via FRET. Furthermore, the AlPcS-QDs killed KB cancer cells under the irradiation of a 532-nm laser with a typical light-dose-dependent PDT mode, demonstrating a new excitation way of FRET-mediated PDT by AlPcS-QDs. The ROS produced by a FRET way of AlPcS-QDs was further examined as the ^1^O_2_. With white light irradiation, the combination of direct and indirect (FRET) excitations for AlPcS-QDs can remarkably enhance PDT effect to cells. In addition, the AlPcS-QD conjugates can easily penetrate into cells, demonstrating that these conjugates are good carriers for AlPcS intracellular delivery because the cellular uptake rate for free AlPcSs is very low. The result also reflects that the positively charged QDs are probably suitable carriers to load negatively charged PSs for intracellular delivery. These results suggest that the new strategy of AlPcS-QD conjugates combined with the FRET could be a feasible modality for PDT, and this new model is worth investigating further to improve PDT effects in cancer treatments.

## Competing interests

The authors declare that they have no competing interests.

## Authors’ contributions

LL carried out the main experimental works. JFZ did the AlPcS' MTT assay in cells. NW and HJ synthesized the QDs. JYC and SK designed the whole work, and JYC organized the manuscript. All authors read and approved the final manuscript.

## Authors’ information

Ji-Yao Chen is a professor and doctorate degree holder in the State Key Laboratory of Surface Physics, Department of Physics, Fudan University. Sungjee Kim is a professor and doctorate degree holder in the Department of Chemistry, Pohang University of Science and Technology. Lei Li and Jin-Feng Zhao are master degree holders in the Department of Physics, Fudan University. Nayoun Won and Ho Jin are PhD candidates in the Department of Chemistry, Pohang University of Science and Technology.
